# Different Persistence of the Cellular Effects Promoted by Protein Kinase CK2 Inhibitors CX-4945 and TDB

**DOI:** 10.1155/2015/185736

**Published:** 2015-10-19

**Authors:** Cristina Girardi, Daniele Ottaviani, Lorenzo A. Pinna, Maria Ruzzene

**Affiliations:** Department of Biomedical Sciences and CNR Institute of Neuroscience, University of Padova, 35131 Padova, Italy

## Abstract

We compare the cellular efficacy of two selective and cell permeable inhibitors of the antiapoptotic kinase CK2. One inhibitor, CX-4945, is already in clinical trials as antitumor drug, while the other, TDB, has been recently successfully employed to demonstrate the implication of CK2 in cellular (dis)regulation. We found that, upon treatment of cancer cells with these compounds, the extent of inhibition of endocellular CK2 is initially comparable but becomes significantly different after the inhibitors are removed from the cellular medium: while in CX-4945 treated cells CK2 activity is restored to control level after 24 h, in the case of TDB it is still strongly reduced after 4 days from removal. The biological effects of the two inhibitors have been analyzed by performing clonogenic, spheroid formation, and wound-healing assays: we observed a permanent inhibition of cell survival and migration in TDB-treated cells even after the inhibitor removal, while in the case of CX-4945 only its maintenance for the whole duration of the assay insured a persisting effect. We suggest that the superiority of TDB in maintaining kinase activity inhibited and perpetuating the consequent effects is an added value to be considered when planning new therapies based on CK2 targeting.

## 1. Introduction

CK2 is a Ser/Thr protein kinase ubiquitously expressed and constitutively active, which phosphorylates hundreds of substrates and is involved in different cellular processes [1, 2]. Its activity is especially relevant for cancer cells [3], which not only express higher amount of CK2 compared to normal cells, but also rely more on it for their survival, being often dependent on tumor-specific prosurvival pathways which are potentiated by CK2 [4]. For these reasons, and despite its expression also in healthy cells, CK2 is presently considered a valuable anticancer therapeutic target. Several CK2 inhibitors have been developed so far [5, 6], but the most promising results have been obtained with CX-4945. This compound, initially discovered by Cylene Pharmaceuticals Inc. [7], is an ATP-competitive inhibitor, which is very selective for CK2 and is presently the only CK2 inhibitor under clinical trials in humans for cancer therapy [8, 9]. CX-4945 displays a very strong efficacy towards CK2 in vitro, with a IC_50_ of 1-2 nM [10]. However, when used to treat cancer cells, the concentrations required to induce apoptosis are not proportionally low; for example, in one of the initial studies, published by Pierre and coworkers [8], the calculated Ki for the CK2 catalytic subunit *α* in vitro was 1 nM, while the concentrations required to induce 50% cell death (DC_50_) varied between 1 and 9 *μ*M. We found very similar results in a study on different pairs of apoptosis sensitive/resistant variants of tumor cell lines [11] (DC_50_ 2–9 *μ*M), and many other similar examples can be found in the literature, from where it is evident that CX-4945 is commonly used in the *μ*M range to inhibit CK2 in cells.

We have recently developed a compound called TDB, which is another ATP-competitive CK2 inhibitor with promising therapeutic features [12]. We found that TDB is more effective than CX-4945 in inducing cell death. Such a superiority was not expected, since the IC_50_ for CK2 of TDB (32 nM) is manyfold higher than that of CX-4945. We therefore performed the present study aimed at analyzing the cellular efficacy of these two compounds. By comparing the persistence of CK2 inhibition after their administration to cells, we found that the effect of TDB on CK2 activity is more long-lasting than that of CX-4945, with important consequences on cellular processes affected by CK2, such as cell survival, proliferation, and migration.

## 2. Materials and Methods

### 2.1. Inhibitors

TDB (previously called K164) was synthesized as described in [13]. CX-4945 was from AbMole Bioscience. Inhibitors solution was in made in DMSO.

### 2.2. Cell Culture and Treatment

All cells were cultured in an atmosphere containing 5% CO_2_; CEM cells (human T lymphoblastoid) were maintained in RPMI 1640 medium (Sigma) and U2OS cells (human osteosarcoma) and HEK-293T (human embryonic kidney cells) in D-MEM medium (Sigma); both media were supplemented with 10% (v/v) fetal calf serum (FCS), 2 mM L-glutamine, 100 U/mL penicillin, and 100 mg/mL streptomycin. Cell treatments with inhibitors were performed in the culture medium. Control cells were treated with equal amounts of the inhibitor solvent, which never exceeded 0.5% (v/v).

### 2.3. Cell Transfection

U2OS cells were transfected with Akt1 plasmid (2 *μ*g) [14], using TransIT (Mirus Bio, Madison, USA) as transfecting agent. After 40 h from the beginning of the transfection, cells were treated with inhibitors or vehicle for 24 h. Then the medium was removed and cells were immediately lysed or cultured for further 24 h in the absence of inhibitors.

### 2.4. Cell Lysis and Western Blot Experiments

For lysate preparation, cells were lysed as described in [15]. Protein concentration was determined by the Bradford method. Equal amounts of proteins were loaded on 11% SDS-PAGE, blotted on Immobilon-P membranes (Millipore), and processed in Western blot (WB) with the indicated antibody, detected by chemiluminescence. Quantitation of the signal was obtained by chemiluminescence detection on a Kodak Image Station 440MM PRO and analysis with the Kodak 1D Image software.

### 2.5. CK2 Activity Assay

CK2 activity in cell lysates was measured by means of radioactive assays with [*γ*-^33^P]ATP towards the specific CK2 substrate peptide CK2-tide, as described in [16].

### 2.6. Clonogenic Survival Assays

U2OS cells were plated at 100 cells/well in 6-well plates and allowed to adhere to the plate for 16 h. Cells were then treated with variable concentrations of the inhibitors (or with equal amount of vehicle as control) in culture medium. After 24 h, cells were washed and the medium was replaced by fresh culture medium without inhibitors. After further 6 days, cells were fixed and stained with 0.5% crystal violet; images were captured using a Leica DMI4000 automated inverted microscope equipped with a Leica DFC300 FX camera.

### 2.7. 3D Spheroid Formation

U2OS cells were plated at 50% confluence, allowed to adhere to the plate for 16 h, and then treated with inhibitors or vehicle for 24 h. After treatment, cells were washed, detached, counted, and seeded at 1000 cells/well in 200 *μ*L of culture medium, in a 96-well plate with round bottom wells precoated with 50 *μ*L of 1% agar in culture medium [17]. Images were taken from each well 24 h and 96 h later, by means of a Leica DMI4000 automated inverted microscope equipped with a Leica DFC300 FX camera.

### 2.8. Wound-Healing Assays

Wound-healing assays were performed by creating identical wound areas into the cell monolayer using Ibidi culture-inserts (Ibidi GmbH, Cat. no. 80209, Munich, Germany). U2OS cells were seeded in complete culture medium at a density of 3 × 10^4^ cells on each side of the Ibidi culture-insert, into a 24-well plate. After attachment, cells were treated for 24 h inhibitors (or vehicle in the controls); then the culture-insert was detached in order to form a cell-free gap into the cell monolayer. Each well was rinsed once with phosphate-buffered saline (PBS) to remove cell debris and immediately refilled with fresh medium, with new addition of the inhibitors (samples with “maintained” treatment) or without (sample with “removed” treatment). Cells were allowed to migrate for further 48 h. The wound images were captured at time zero (*t* = 0 h), *t* = 24 h, and *t* = 48 h using a Leica DMI4000 automated inverted microscope equipped with a Leica DFC300 FX camera.

## 3. Results

**Figure 1:**
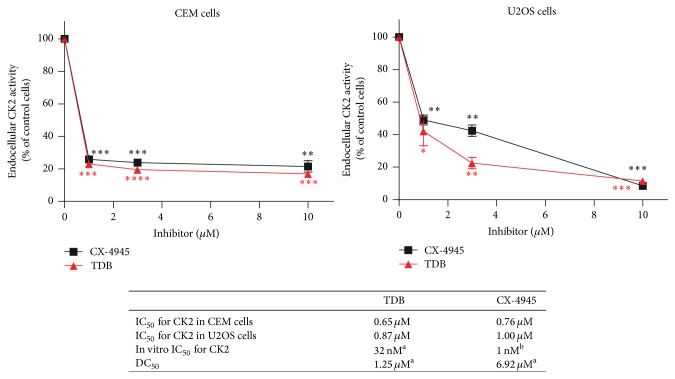
Comparison of CX-4945 and TDB efficacy on cellular CK2 activity. CEM or U2OS cells were treated for 4 h with increasing concentrations of TDB or CX-4945. Then cells were lysed and CK2 activity was assessed from 1-2 *μ*g of lysate proteins towards a synthetic CK2-specific peptide. Activity is shown as percent of control vehicle-treated cells (mean of two independent experiments ± SEM). In the box, the IC_50_ values for CK2 inhibition in the two cell lines are reported, as extrapolated from the curves above; the in vitro IC_50_ values for CK2 are also shown for comparison, as reported in [12] (^a^) (SEM never exceeding 10%) and [7] (^b^). DC_50_ values (concentrations required to induce 50% of cell death in 24 h) are reported as in [12] (^a^) for HeLa cells, where a direct comparison between the two compounds in inducing cell death is presented. Statistical significance was calculated using unpaired *t*-test between control and treated cells (^*∗∗∗∗*^ *p* ≤ 0.0001; ^*∗∗∗*^ *p* ≤ 0.001; ^*∗∗*^ *p* ≤ 0.01; ^*∗*^ *p* ≤ 0.05).

**Figure 2:**
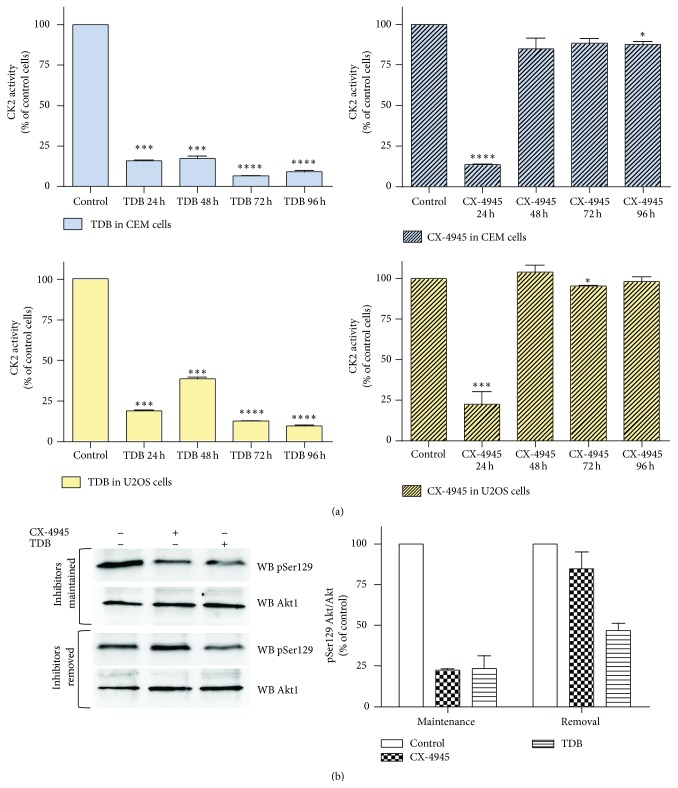
Duration of CK2 inhibition after TDB or CX-4945 removal from treated cells. CEM or U2OS cells were treated with TDB or CX-4945, as indicated, for 24 h. Then cells were washed and the culture medium was replaced with a fresh one devoid of inhibitors. (a) Cells were cultured for a further time, as indicated, and then lysed. CK2 activity was assessed from 1-2 *μ*g of lysate proteins towards a synthetic CK2-specific peptide. The inhibitor concentration was 3 *μ*M. Activity is shown as percent of control vehicle-treated cells (mean of three independent experiments ± SEM; statistical significance was calculated using unpaired *t*-test between control and treated cells (^*∗∗∗∗*^ *p* ≤ 0.0001; ^*∗∗∗*^ *p* ≤ 0.001; ^*∗∗*^ *p* ≤ 0.01; ^*∗*^ *p* ≤ 0.05)). (b) Cells were lysed after the first 24 h of culture in the presence of 10 *μ*M inhibitors (upper blots) or cultured for further 24 h after the inhibitor removal (lower blots). 10 *μ*g of lysate proteins was analyzed by WB with the indicated antibodies. A quantification of the pSer129 (mean of three independent experiments ± SEM) normalized to Akt1 total amount is shown in the histograms on the right, where 100% values have been assigned to untreated cells of each experimental protocol.

To compare the inhibitory efficacy in cells of the two compounds, CX-4945 and TDB, we analyzed their effects on two cell lines, one deriving from a blood tumor (CEM, T-cell lymphoblastoma) and the other from a solid tumor (U2OS, osteosarcoma). Their efficacy was quite similar in inhibiting cellular CK2 (Figure 1). However, based on their different in vitro IC_50_ values towards CK2 (also shown in Figure 1, box), a much stronger efficacy of CX-4945 was expectable. Moreover, the ability of TDB to induce cell death is higher than that of CX-4945 (see DC_50_ values in the box of Figure 1, calculated from [12]), suggesting that TDB has peculiar features that render it more effective than expected. We therefore decided to assess whether the permanence of CK2 inhibition in cells induced by the two compounds was different. To this purpose, we performed experiments of cell treatment for 24 h with the inhibitors, followed by cell washing and replacement of a fresh, inhibitor-free, medium; lysates from treated cells were then analyzed for CK2 activity. In this kind of experiments we observed that, in cells treated with CX-4945, CK2 activity was rapidly restored to control level, while in the case of TDB the effect was much more persistent, lasting up to 4 days after the inhibitor removal (Figure 2(a)). To address this point by a different approach allowing direct measuring of the endocellular CK2 activity, we transfected U2OS cells with Akt1, whose Ser129 works as a reporter of CK2 activity in cells [14, 18]. The results, shown in Figure 2(b), demonstrated that the phosphorylation of Akt1 Ser129 is similarly reduced by the two inhibitors immediately after treatment, while it remains significantly lower than the control only with TDB in case of inhibitor removal, thus confirming the more permanent blockage of the kinase activity by TDB than by CX-4945.

**Figure 3:**
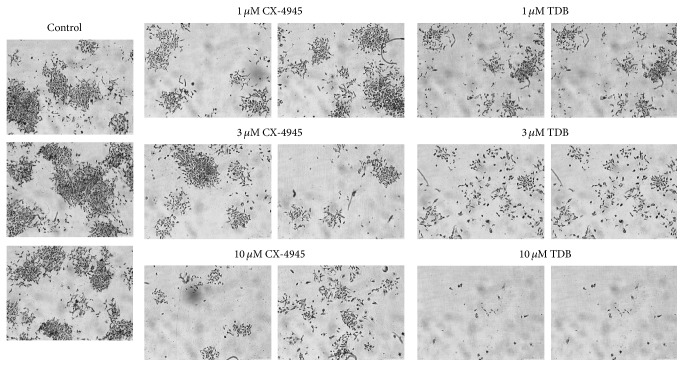
Effects of CX-4945 and TDB on clone formation and survival. 100 U2OS cells were plated for each well, treated for 24 h with the indicated inhibitors, and then grown for further 6 days in the absence of the inhibitors. Two representative images are shown for each condition (2.5x objective, magnification-changer 1.6); at least two separate experiments in triplicate were performed.

**Figure 4:**
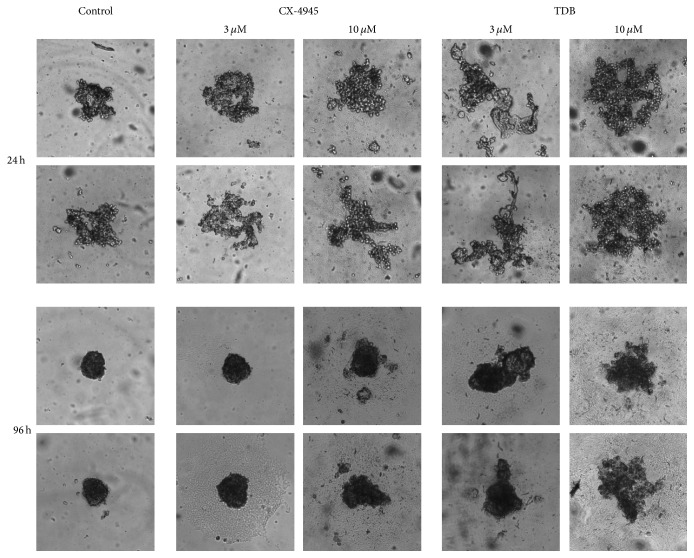
Effects of CX-4945 and TDB on spheroid formation. U2OS cells were pretreated for 24 h with the indicated concentrations of inhibitors and then allowed to form spheroids. Images were taken after 24 h and 96 h. Two separate experiments were performed, with triplicates of each condition (2.5x objective, magnification-changer 1.6). Two representative images for each condition are shown.

We wondered if the different duration of CK2 inhibition by CX-4945 and TDB had consequences on cellular processes controlled by CK2. To assess this point, we first performed clonogenic survival assays in U2OS cells, by seeding cells in 6-wells plates, treating with increasing concentrations of CX-4945 or TDB for 24 h, then removing the medium, and allowing clones to grow for further 6 days in the absence of the inhibitors. The results, shown in Figure 3, indicate that TDB is much more effective than CX-4945 in preventing clone formation and survival. Similar results were obtained in 3D culture experiments to assess spheroid formation (Figure 4): we found that TDB-treated cells were much less prone than vehicle-treated cells to forming spheroids, while CX-4945 was similarly effective than TDB when assessed at 24 h but has much weaker effect at longer times after the inhibitor removal.

**Figure 5:**
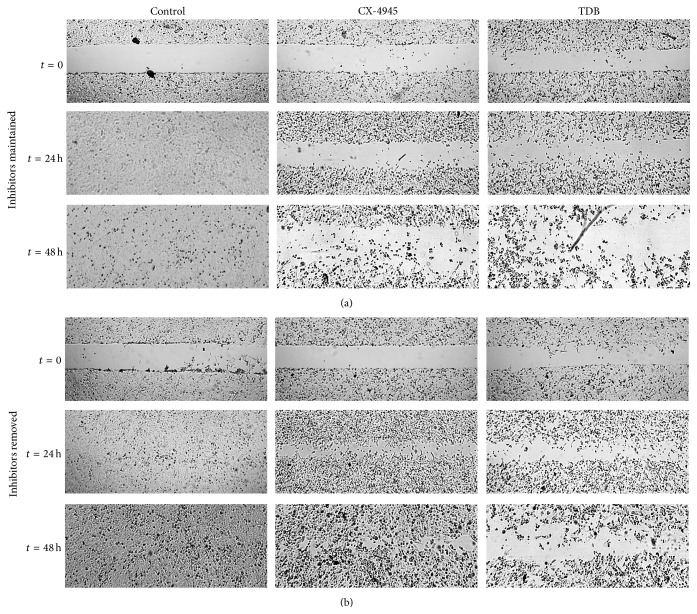
Effects of CX-4945 and TDB on cell migration. Cell migration was assessed by wound-healing assay, seeding U2OS cells in wells containing an insert, and incubating them for 24 h in the presence of the indicated inhibitor (10 *μ*M) or vehicle. Then the insert was removed (*t* = 0) and cells were left to migrate for 48 h, always in the presence of the inhibitors (upper part of the figure, “inhibitors maintained”) or in their absence (lower part of the figure, “inhibitors removed”). The experiment was performed three times, with each condition in duplicate. Representative images at *t* = 0, *t* = 24 h, and *t* = 48 h are shown.

We then evaluated U2OS cells for their migration activity in wound-healing assays in the presence of the two compounds.  Figure 5 shows that, as expected for CK2 inhibitors [19–21], both CX-4945 and TDB were able to inhibit cell motility in 24–48 h assays when cells were constantly exposed to them (upper part of the figure). By contrast, when the inhibitors were removed from the medium after a 24 h treatment (before starting the analysis of cell migration, *t* = 0 in Figure 5), cells migrated significantly less than the control cells only in the case of TDB (lower part of Figure 5).

All our observations unequivocally indicate that the effect of TDB persists in the cells more than that of CX-4945, suggesting more stable binding of the former to the target kinase.

## 4. Discussion

Theoretically, the ATP-competitive compounds work as kinase inhibitors by a reversible mechanism, relying on noncovalent interactions with the ATP pocket of the kinase. In the case of both CX-4945 and TDB, this assumption has been validated by crystallographic studies [10, 12]. Intriguingly, however, many of them are fully active at *μ*M concentrations (or below) even in the cellular environment, where the ATP concentration, in the mM range, would be expected to vanish their efficacy. This can be explained in terms of affinity, considering that the Km values of kinases for ATP are in the *μ*M range, while many inhibitors display Ki values in the low nM range. The possibility, however, that the occupancy of the ATP site is stabilized by additional mechanisms should be also considered. Different inhibitors may display for the same kinase similar affinity but sharply different dissociation rates, thus leading to residence times that are strikingly variable, from few minutes to several hours. This concept may also contribute to account for the observation that the ratio between DC_50_ (reflecting in cell efficacy) and IC_50_ (measured in vitro) is also very variable depending on the inhibitor considered. With this in mind we performed this study, focused on two inhibitors that induce cellular effects at concentrations that are not proportional to their inhibitory potency in vitro. One of them, CX-4945, is considered quite selective for CK2, while the other, TDB, is a dual inhibitor, targeting also PIM-1, another antiapoptotic kinase, which is often overexpressed in cancer cells [22]. However, the observation that TDB is more effective than CX-4945 in cells cannot be simply explained by the dual nature of TDB, since the combination of a CK2 and a PIM-1 inhibitor is still less effective than TDB [12]. Moreover, although CX-4945 is considered a pure CK2 inhibitor, it reduces PIM-1 activity as well: its IC_50_ for PIM-1 (216 nM [10]) is comparable to that of TDB (86 nM [12]). Consistently, the residual activity of PIM-1 in the presence of 0.5 *μ*M CX-4945 is only 6% [10], while it is 7% in the presence of 1 *μ*M TDB [12]. At the concentrations used in cells, therefore, it is conceivable that both inhibitors target PIM-1 besides CK2, suggesting that TDB superiority should rely on other features, and that it possess a sort of added value compared to other inhibitors. Here we show that the most relevant difference is the duration of CK2 inhibition, since in CX-4945 treated cells CK2 activity is rapidly restored to basal levels after the removal of the inhibitor, while in TDB-treated cells the inhibition lasts for several days (Figure 2). This feature was found in two different cell lines, one from hematological and one from solid tumor. We can not exclude that the cellular environment can affect the stability of the kinase/inhibitor complex in a cell-specific manner. Indeed, when we performed this kind of experiments on HEK-293T (nontumor cells), we found that the inhibition by CX-4945 was transient (13 ± 3% of residual CK2 activity after 24 h treatment with 10 *μ*M CX-4945, 83 ± 0.5% after further 24 h in the absence of the inhibitor), similarly to what observed in tumor cells, while the effect of TDB was slightly more persistent (17.9 ± 0.9% residual activity after 24 h with 10 *μ*M TDB, 55.5 ± 5.5% after further 24 h in the absence of the inhibitor), albeit less than in the tumor cells that we analyzed.

For the time being, what we can say is that in our cancer models this different property of the two inhibitors has important consequences on cellular processes. As expected, in fact, longer persistence of CK2 inhibition is more effective in reducing the clonogenic survival of tumor cells (Figure 3), their ability to form spheroids (Figure 4), and their migration (Figure 5).

The exact reason for the longer inhibition induced by TDB compared to CX-4945 will deserve future investigation. Here we hypothesize that a possible difference between the two compounds could be related to the stability of their complex with CK2, since the experiments have been performed in conditions of inhibitor removal. However, other possibilities should be also taken into account. In accordance with our results, Schwind and colleagues have recently published a paper [23] where the inhibition by CX-4945 and its effects on stem cell differentiation were found to decrease from 24 h to 72 h. Since in that case the inhibitor was maintained in contact with cells for all the length of the experiment, the result would indicate that its effective concentration decreased, and this could be due to several causes, such as compound extrusion, subcellular redistribution, or metabolic inactivation.

CK2 being considered a valuable therapeutic target [4, 24, 25], our results can be relevant also from a therapeutic point of view. A part from a chimeric peptide called CIGB-300 [26] which is giving promising results on cervical carcinoma [27] but whose mechanism of action is still enigmatic [28], CX-4945 is the only ATP-competitive small inhibitor of CK2 in phase II of clinical trials as anticancer agent [8]. In the comparison presented here, however, TDB is superior to CX-4945. Taking also into account that, besides CK2, it inhibits PIM-1 (a very important and innovative target in cancer therapy [29–31]), CLK2, and, with lower efficacy, also DYRK1A [12], we believe that TDB displays good features to be considered for future clinical experimentation.

In this work we focused on CK2 activity. However, we cannot exclude that the same persistence of kinase inhibition by TDB observed on CK2 can also occur on PIM-1 and the other major targets of TDB. Future studies will be required to address this point; for the time being, irrespective of the kinase affected and the molecular mechanism, we can conclude that survival, proliferation, and migration of tumor cells are more stably affected by TDB than by CX-4945.

## 5. Conclusions

In this paper we highlight an important difference between the two CK2 inhibitors, CX-4945 and TDB, consisting in a different duration of their effects in cells, which is longer for TDB. This feature was unpredictable from the in vitro efficacy of the two inhibitors towards protein kinase CK2, which instead indicated a superiority of CX-4945. Our findings suggest that the persistence of cellular effects should be therefore considered as an additional evaluation element to be taken into account in investigations on CK2 inhibitors as potential therapeutic tools.

## Abbreviations

CK2: Casein kinase 2
CX-4945: 5-(3-Chlorophenylamino)benzo[c][2,6]naphthyridine-8-carboxylic acid
DC_50_: Concentrations required to induce 50% of cell death in 24 h
DMSO: Dimethyl sulfoxide
PBS: Phosphate-buffered saline
PIM-1: Proviral integration of Moloney virus
TDB: Tetra-bromo-deoxyribofuranosyl-benzimidazole
WB: Western blot.
